# Preparation and In Vitro and In Vivo Evaluation of Rectal In Situ Gel of Meloxicam Hydroxypropyl-β-cyclodextrin Inclusion Complex

**DOI:** 10.3390/molecules28104099

**Published:** 2023-05-15

**Authors:** Xiaomeng Lei, Guansheng Zhang, Tao Yang, Yuhuan Wu, Ying Peng, Tiantian Wang, Dongxun Li, Qian Liu, Canjian Wang, Guosong Zhang

**Affiliations:** 1 National Engineering Research Center of Chinese Medicine Solid Preparation Manufacturing Technology, Jiangxi University of Chinese Medicine, Nanchang 330006, China; 2 Integrated Chinese and Western Medicine Institute for Children Health & Drug Innovation, Jiangxi University of Chinese Medicine, Nanchang 330006, China; 3 College of Chinese Medicine and Life Science, Jiangxi University of Chinese Medicine, Nanchang 330004, China

**Keywords:** meloxicam, thermosensitive in situ gel, HP-β-CD inclusion, rectal delivery, bioavailability

## Abstract

Meloxicam (MLX) is one of the most effective NSAIDs, but its poor water solubility and low bioavailability limit its clinical application. In this study, we designed a thermosensitive in situ gel of the hydroxypropyl-β-cyclodextrin inclusion complex (MLX/HP-β-CD-ISG) for rectal delivery to improve bioavailability. The best method for preparing MLX/HP-β-CD was the saturated aqueous solution method. The optimal inclusion prescription was optimized using an orthogonal test, and the inclusion complex was evaluated via PXRD, SEM, FTIR and DSC. Then, MLX/HP-β-CD-ISG was characterized regarding the gel properties, release in vitro, and pharmacokinetics in vivo. The inclusion rate of the inclusion complex obtained via the optimal preparation process was 90.32 ± 3.81%. The above four detection methods show that MLX is completely embedded in the HP-β-CD cavity. The developed MLX/HP-β-CD-ISG formulation has a suitable gelation temperature of 33.40 ± 0.17 °C, a gelation time of 57.33 ± 5.13 s, pH of 7.12 ± 0.05, good gelling ability and meets the requirements of rectal preparations. More importantly, MLX/HP-β-CD-ISG significantly improved the absorption and bioavailability of MLX in rats, prolonging the rectal residence time without causing rectal irritation. This study suggests that the MLX/HP-β-CD-ISG can have a wide application prospect with superior therapeutic benefits.

## 1. Introduction

**Figure 1:**
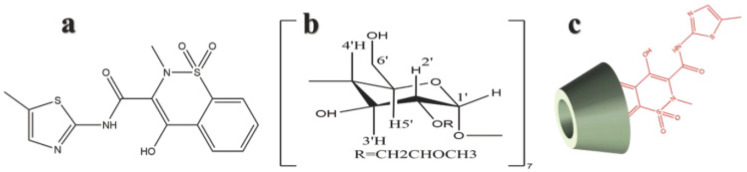
Chemical structures of MLX (**a**), HP-β-CD (**b**) and MLX/HP-β-CD inclusion complex (**c**).

Meloxicam (MLX) is a novel enolamide non-steroidal anti-inflammatory analgesic drug (NSAID) [1] (Figure 1a) and a selective inhibitor of cyclooxygenase-2 (COX-2) [2], mainly used in the treatment of osteoarthritis and rheumatoid arthritis [3]. Compared with opioid analgesics, it presents a lower chance of gastrointestinal reactions and fewer side effects. However, according to the Biopharmaceutical Classification System (BCS), it is classified as a class II drug with low solubility(4.4 µg/mL) and high permeability, which greatly limit its bioavailability [4,5]. Therefore, its low bioavailability caused by low water solubility limits a wide range of applications for MLX.

In recent years, cyclodextrin inclusion technology has achieved good results in improving the solubility, chemical stability and bioavailability of hydrophobic drugs [6,7,8]. Bandarkar et al. prepared a stable inclusion complex of MLX/β-CD with enhanced aqueous solubility and dissolution rate using a highly efficient and controlled milling technique [9]. Samprasit et al. prepared MLX/2-HP-β-CD oral dissolving films. The results showed that the MLX/2-HP-β-CD complexes improved the solubility of MLX [10]. Akkaramongkolporn et al. developed orally disintegrating tablets using a combination of ion exchange resin and HP-β-CD. The results showed that MLX and HP-β-CD demonstrated complete solubility and significant stability [11]. Rein et al. investigated an in situ forming system based on MLX in β-CD for periodontitis treatment. The developed system, comprising 40% β-CD transformed into microparticles, extended the drug release to 7 days in the locality of the treatment [12]. Jafar et al. improved the aqueous solubility and dissolution rate of meloxicam by preparing its ternary complex with β-cyclodextrin and triethanolamine. The solubility, stability and anti-inflammatory activity of meloxicam were successfully increased [13]. Ainurofiq et al. investigated the inclusion complexes of MLX/β-CD incorporated into orally disintegrating tablets with excellent drug release and solubility enhancements [14]. Shende investigated the inclusion complexes of meloxicam and β-CD-based nanosponges to enhance their solubility and stability and prolong release [15]. In conclusion, CDs are promising drug delivery systems capable of improving the solubility and chemical stability of MLX.

Considering that most NSAID preparations aim for systemic distribution, the development of pharmaceutical formulations based on CD inclusion complexes may be a promising strategy to reverse their low solubility [16,17]. The rectal delivery is an interesting alternative to the oral route, decreasing systemic side effects and avoiding first-pass metabolism [18]. Balakrishnan et al. prepared a Clotrimazole/β-CD suppository, and the results showed that the prepared inclusion complex suppository could be a potential suppository formulation to increase the bioavailability of hydrophobic drugs, such as clotrimazole [19]. However, the suppository is not easy to retain in the body and flows easily from the rectum after softening. It can also gradually reach the end of the colon automatically, resulting in a liver first-pass effect. A rectal in situ gel is a specific dosage form for local application in the intestine [20]. It is administered in a solution state. Under the stimulation of the physiological environment, the phase transition occurs immediately at the application site, and the liquid transformation creates a non-chemical, cross-linked semi-solid gel [21]. It has appropriate gelling strength and is bioadhesive; it does not leak easily from the anus and does not move upward. It can be retained locally and has long-term efficiency at the local level [22]. Wang et al. formed an inclusion complex of 5-FU and cyclodextrin and then embedded it in the in situ gel matrix. The results showed that the solubility of 5-FU was increased, the release of 5-FU was promoted, the release time was prolonged, and the problem of low bioavailability was also solved [23].

In order to improve the problems of the poor solubility of MLX, the easy movement of the solid suppository and the easy outflow of the enema solution, two new preparation techniques are used in this paper. Combined with cyclodextrin (Figure 1b) encapsulation technology (Figure 1c) and temperature-sensitive polymer materials, MLX was embraced into an inclusion compound and then loaded into an in situ gel, which was evaluated in vivo and in vitro. It enables MLX to achieve the purpose of special administration and improve drug efficacy by enhancing solubility. The MLX/HP-β-CD-ISG prepared in this experiment reached the rectum in the form of a solution and turned into a gel state at rectal temperature. As a compound drug delivery system, it has dual characteristics of the CD inclusion complex and an in situ gel, which improve the solubility of MLX, enhance the permeability and prolong the residence time of MLX in the rectum. It also overcomes the problems of the liver first-pass effect and eases the leakage of traditional suppositories and enemas and provides a new preparation for rectal administration.

## 2. Results and Discussion

### 2.1. Preparation of the Inclusion Complex of the MLX/HP-β-CD

The formation of the inclusion complex is the process of drug molecules entering the molecular cavity of the inclusion material. In this process, a certain amount of energy needs to be provided to promote full contact between the drug and the inclusion material, and for the drug molecules to slowly enter the molecular cavity of the inclusion material. Different preparation methods provide different energy amounts, so the preparation method has an important influence on the formation of inclusion complexes. Therefore, two methods were used to prepare MLX/HP-β-CD inclusion complexes, and two methods were screened with MLX inclusion efficiency as the evaluation index. The results show that the inclusion efficiency of MLX in the inclusion complexes obtained by the two methods were 84.39 ± 1.52% and 90.61 ± 0.98%. The better inclusion efficiency was obtained by the saturated solution method, so this method was selected to prepare the inclusion complex in this study.

### 2.2. Optimization of the Preparation Method of the MLX/HP-β-CD Complexes

The results of the orthogonal test (Tables S1 and S2, Supplementary Materials) showed that the optimal preparation process of the MLX/HP-β-CD inclusion complex was A_3_B_3_C_2_D_3_, and the ratio of MLX to HP-β-CD had the greatest influence on the inclusion effect. Therefore, the optimal process was as follows: the ratio of MLX to HP-β-CD was 1:8, the amount of ammonia was 15 mL, the inclusion time was 60 min, the inclusion temperature was 40 °C and the inclusion rate was 90.32 ± 3.81%.

### 2.3. Characterization of the MLX/HP-β-CD Inclusion Compounds

#### 2.3.1. Solubility

The average solubilities of MLX and the MLX/HP-β-CD inclusion complex in pure water were 13.5 µg/mL and 66 µg/mL, respectively. The results showed that the solubility of the MLX/HP-β-CD inclusion complex was 4.9 times higher than that of MLX.

#### 2.3.2. Phase Solubility

**Figure 2:**
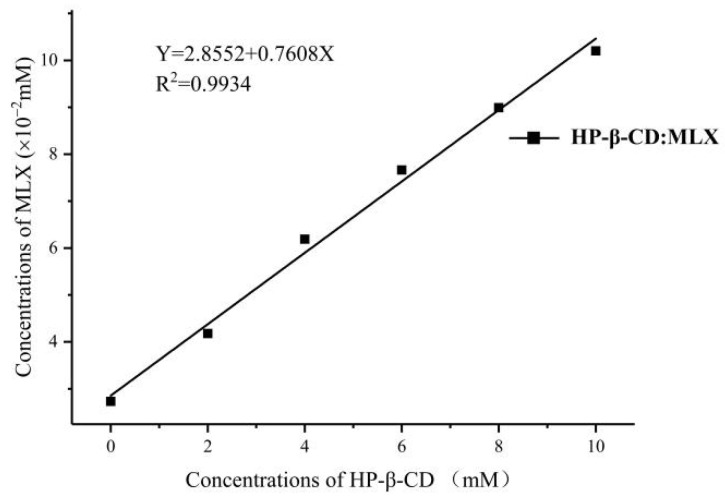
Phase solubility study of the MLX/HP-β-CD inclusion complex.

Figure 2 shows the phase solubility diagram of MLX and HP-β-CD. It can be seen that the solubility of MLX increases with the increase in HP-β-CD concentration. In this experimental process, it had a linear relationship with the HP-β-CD concentration, which was an AL-type curve; the regression equation obtained was Y = 2.8552 + 0.7608X (R^2^ = 0.9934) and the slope value (0.7608), defined in the phase solubility diagrams, was less than a unit for HP-β-CD, indicating that the inclusion complex stoichiometry of MLX and HP-β-CD was 1:1. The apparent stability constants (K_1:1_) can be calculated by formulas for MLX/HP-β-CD inclusion compounds from phase solubility diagrams according to the following Equation (1):

(1) $$K_{1:1}=\frac{slope}{S_{0}(1-slope)}$$

where *S*_0_: the intrinsic solubility of MLX.

The stability constant value calculated was 280.30 M^−1^. It has been reported that the K_1:1_ value for a stable complex is within the range of 100–1000 M^−1^ [24]. Correspondingly, K_1:1_ calculated using *S*_0_ suggested that HP-β-CD produces stable inclusion complexes with MLX.

#### 2.3.3. Differential Scanning Calorimetry (DSC)

**Figure 3:**
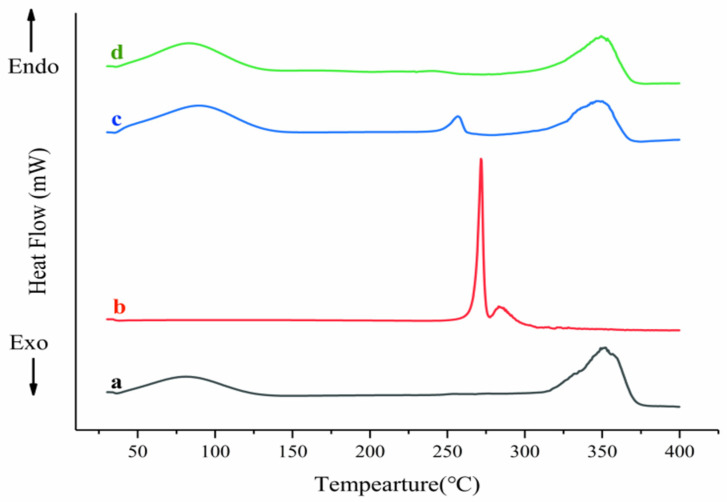
DSC patterns of HP-β-CD (**a**), MLX (**b**), physical mixture (**c**) and the inclusion complex (**d**).

DSC is a very common technique for verifying the formation of inclusion complexes. When the drug molecules are partially or completely encapsulated into the HP-β-CD cavity, the melting point, boiling point and sublimation point of the drug will move to different temperatures or even disappear. It can be seen from Figure 3b that MLX had a single sharp melting endothermic peak at about 271 °C, corresponding to the melting temperature of MLX [25]. Compared to MLX, HP-β-CD (Figure 3a) had an endothermic peak of around 75 °C and a complex endothermic peak from 300 to 370 °C, which corresponded with water dissociation from the compound and the melting point and decomposition of HP-β-CD, respectively [26]. In the physical mixture of MLX and HP-β-CD (Figure 3c), there were both endothermic peaks of MLX and HP-β-CD, indicating that there was almost no interaction between the two components. Meanwhile, the endothermic peak of MLX shifted to a lower temperature, indicating a partial inclusion of MLX in the HP-β-CD cavity. However, the DSC thermograms of the inclusion complex (Figure 3d) were similar to HP-β-CD. The melting point peaks of MLX disappeared, suggesting the success of the inclusion complex formation process. The difference in DSC thermograms suggested the formation of the inclusion complex MLX/HP-β-CD.

#### 2.3.4. Scanning Electron Microscope (SEM)

**Figure 4:**
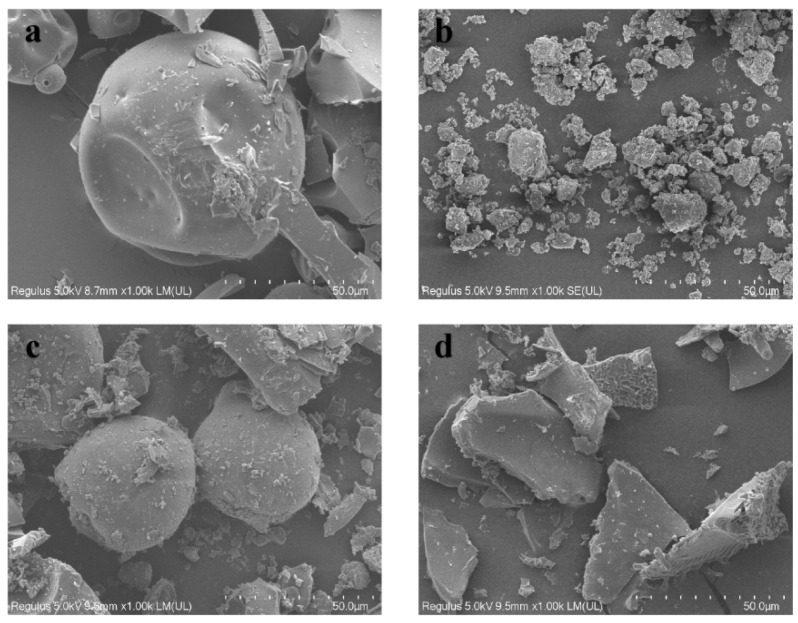
SEM images of HP-β-CD (**a**), MLX (**b**), physical mixture (**c**) and the inclusion complex (**d**).

The crystal size and surface morphology of HP-β-CD (Figure 4a), MLX (Figure 4b), their physical mixture (Figure 4c) and MLX/HP-β-CD inclusion complex (Figure 4d) were observed by scanning electron microscopy. The results show that MLX had a geometric crystal structure, while the surface of HP-β-CD presents a spherical structure with many holes. Compared with the MLX powder, the structure of the inclusion complex had a significant change, showing an irregular sheet structure, which indicated that there was a force between MLX and HP-β-CD. In their physical mixture, the structures of MLX and HP-β-CD could be seen, respectively, indicating that there was a lack of interaction between them. Therefore, MLX had been successfully encapsulated into the HP-β-CD cavity.

#### 2.3.5. Powder X-ray Diffraction (PXRD)

**Figure 5:**
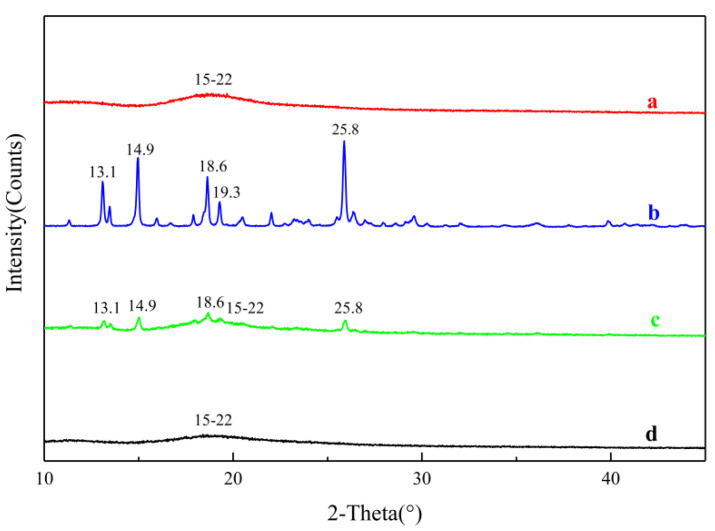
XRD patterns of HP-β-CD (**a**), MLX (**b**), physical mixture (**c**) and the inclusion complex (**d**).

The X-ray diffraction patterns of MLX, HP-β-CD, MLX/HP-β-CD inclusion complex and the physical mixture of MLX and HP-β-CD are shown in Figure 5. The diffraction pattern of MLX had several specific crystal diffraction peaks, while the diffraction pattern of HP-β-CD had no obvious crystal peak, indicating that cyclodextrin existed in an amorphous form. For the physical mixture of MLX and HP-β-CD, the diffraction pattern had both the amorphous state of HP-β-CD and multiple diffraction peaks of MLX, but the peak intensity was significantly weakened or disappeared, which may be due to the dilution of HP-β-CD and MLX after mixing. This showed that there was no new crystal formation between the physical mixtures, and the two were simply physical mixtures. Compared with MLX and HP-β-CD, the diffraction pattern of the MLX/HP-β-CD inclusion complex was similar to that of HP-β-CD, and the characteristic peak of MLX almost disappeared. The crystal form of MLX disappeared because MLX was encapsulated in the cavity of HP-β-CD.

#### 2.3.6. Fourier Transform Infrared Spectroscopy (FTIR)

**Figure 6:**
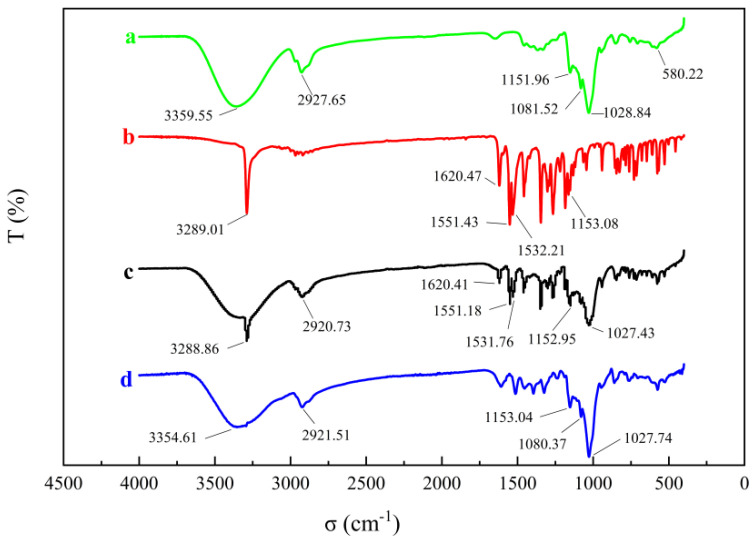
FT-IR spectra of HP-β-CD (**a**), MLX (**b**), physical mixture (**c**) and the inclusion complex (**d**).

The infrared spectra of MLX, HP-β-CD, MLX/HP-β-CD inclusion complexes and their physical mixture are shown in Figure 6. In the infrared spectrum of HP-β-CD, the characteristic absorption band of 3359 cm^−1^ was derived from the vibration of the hydrogen bonds of the hydroxyl group. Some strong absorption peaks appeared at 2927 cm^−1^ (caused by the stretching vibration of CH and CH_3_); 1648 cm^−1^ was the bending vibration of H–O–H, 1151 cm^−1^ was the stretching vibration of C–O and 1028 cm^−1^ was caused by the stretching vibration of the glucose unit C–O–C [27]. The infrared spectrum of MLX showed the presence of the following peaks: 3289 cm^−1^ (secondary CO–NH), 1620 cm^−1^ (CO–NH) and 1551 cm^−1^, 1532 cm^−1^ (N–H bending), 1153 cm^−1^ (SO_2_) and the characteristic absorption band of 3289 cm^−1^ came from the vibration of O–H [28]. In the infrared spectrum of the physical mixture, the characteristic peaks of MLX and HP-β-CD appeared, which indicated that the interaction between the mixtures was very weak or had no interaction. For the inclusion complex, the characteristic absorption peak of MLX at 3289 cm^−1^ (O–H stretching vibration) completely disappeared, which might be due to the co-occurrence of the N–H band and the O–H–intensified band at 3359 cm^−1^, indicating that MLX entered the lipophilic cavity of HP-β-CD. This indicated that there was an interaction between MLX and HP-β-CD. The intensities of the bands appearing at 1620, 1551, 1531 and 1153 cm^−1^ were also affected due to this type of interaction. In summary, through the study of the FTIR of HP-β-CD, MLX, MLX/HP-β-CD inclusion complexes and their physical mixtures, it was shown that MLX was embedded in the cavity of HP-β-CD, and MLX/HP-β-CD inclusion complexes were successfully prepared.

### 2.4. Investigation of the Ratio of P407 and P188

The gelation temperature decreased with the increase in P407 concentration. When the concentration was 20%, the gelation temperature was 25.4 °C, and the gel solution could be transformed into gel at room temperature. With the increase in P188 concentration, the gelation temperature gradually increased. When the concentration reached 6%, the gelation temperature reached 35.7 °C. If the concentration continued to rise, it would be difficult for the gel solution to transform into a gel at body temperature. The gelation temperature of the rectal in situ gel should be close to the rectal temperature, and the human rectal temperature is from 29 to ~34 °C. Therefore, P407 was set to 18%, P188 was set to 4% and the gelation temperature was about 33 °C. The composition of the in situ gel of the MLX/HP-β-CD inclusion complex in situ gel was P407 at 18%, P188 at 4%, HP-β-CD inclusion complex at an appropriate amount and water added to the full amount.

### 2.5. Characterization of MLX/HP-β-CD-ISG

#### 2.5.1. Measurement of the Gelation Temperature and Gelation Time

The gelation of the gel determines the accuracy of the dosage. The gelation temperature should be consistent with the rectal temperature. The shorter the gelation time, the slower the drug loss, and the less likely it is that the burst release reaction occurs. The gelation temperature was 33.40 ± 0.17 °C and the gelation time was 57.33 ± 5.13 s.

#### 2.5.2. Measurement of the Gel Strength

Gel strength is one of the most important indexes of an in situ gel. The gel must have an appropriate strength to be retained at the administration site for a long time, increasing the rectal absorption time, and thus improving bioavailability. Due to the 35 g weight, the preparation did not move in the gel case. The gel strength of MLX/HP-β-CD-ISG was measured to be 50~55 g by changing the weight of the instrument.

#### 2.5.3. Measurement of the Viscosity

The viscosity of MLX/HP-β-CD-ISG at 25 °C was about 400 mPa·s, and the gel viscosity at 37 °C was about 3900 mPa·s. Preparations with a higher viscosity increase the residence time of the drug in the rectum, thereby enhancing drug absorption and reducing the possibility of burst release.

#### 2.5.4. Measurement of pH

The pH value of MLX/HP-β-CD-ISG was directly measured using a pH meter. The pH value was 7.12 ± 0.05, and there was no need to add a pH regulator, as it met the requirements of pH 7~8 for rectal administration [29].

#### 2.5.5. Measurement of the Rheological Properties

**Figure 7:**
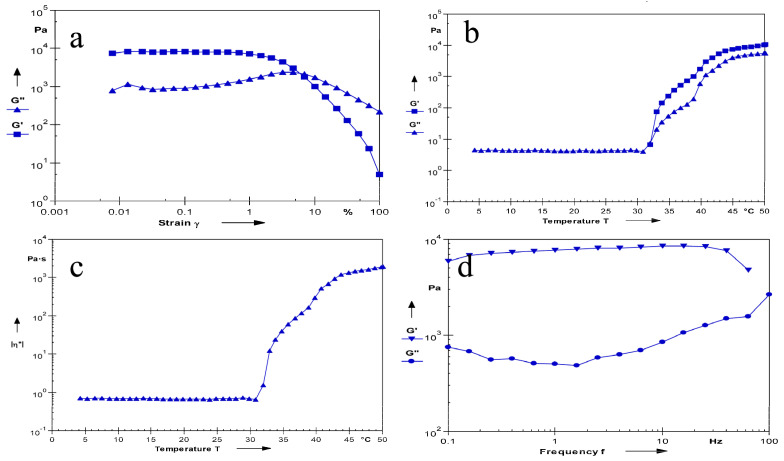
Rheological evaluation of MLX/HP-β-CD-ISG. Linear viscoelastic interval diagram of MLX/HP-β-CD-ISG (**a**); Dynamic temperature scanning diagram of MLX/HP-β-CD-ISG (**b**); Scanning diagram of the composite viscosity changing with the temperature of MLX/HP-β-CD-ISG (**c**); Frequency scanning curves of MLX/HP-β-CD-ISG (**d**).

The determination results of the linear viscoelastic zone of the sample are shown in Figure 7a. It can be seen from the figure that, if the strain’s γ < 1%, the G′ (storage modulus) > G″ (loss modulus), and it is elastic, that is, the sample had a gel structure, when γ > 1%, G′ starts to decline. When G′ = G″, the gel structure is completely destroyed. When G′ < G″, viscosity is prevalent, showing fluid properties. Therefore, all dynamic oscillation experiments were controlled so that γ was within 1%. The results of the phase transition temperature are shown in Figure 7b. It can be seen that G′ and G″ intersect at about 33 °C, and the intersection point is its phase change temperature. Before this temperature, the sample mainly had a loss modulus, showing obvious fluid properties. After the phase change temperature, G′ > G″ gradually indicates that the gel structure is forming, and G′ and G″ tend to be flat, which proves that the viscoelasticity of the gel is stable. It can be seen from Figure 7c that the composite viscosity of the sample suddenly changes at about 33 °C, and the sudden change point is the phase change temperature of the gel, which is consistent with the intersection point of G′ and G″. The frequency scanning results are shown in Figure 7d. In the whole frequency scanning range, the G′ curve is always higher than the G″ curve, showing obvious elasticity-based characteristics, and the G′ is relatively stable without significant frequency dependence, indicating that the gel always has a stable three-dimensional network structure.

#### 2.5.6. Measurement of the Stability

The results (Table S4, Supplementary Materials) showed that the content of MLX/HP-β-CD-ISG decreased at a high temperature (40 °C). It is stable under high humidity and high-intensity light conditions. Therefore, the preparation should be stored at a low temperature. So MLX/HP-β-CD-ISG was suitable for low-temperature storage.

### 2.6. In Vitro Drug Release Study

**Figure 8:**
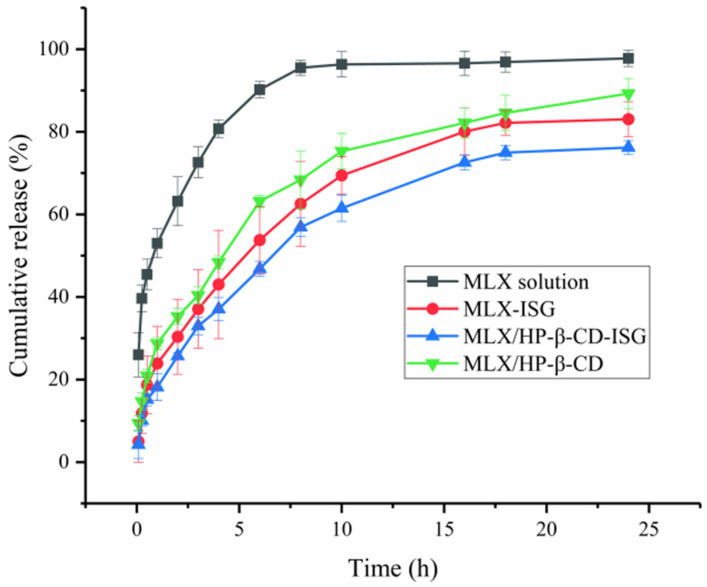
In vitro drug release profiles of MLX from different formulations in phosphate buffer (The data in the graph is represented as X ± SD, *n* = 4).

In vitro release is shown in Figure 8. The release of MLX from MLX/ HP-β-CD-ISG was longer than that of MLX-ISG, MLX/HP-β-CD and MLX solution, and the % cumulative MLX release at 10 h was 60.32 ± 2.59%. At the same time, MLX/HP-β-CD reached 78.31 ± 4.33% and MLX-ISG reached 69.43 ± 4.58% cumulative MLX release. However, the MLX solution basically reached this release degree in around 4 h, and 6h released 85%, almost completely released. This showed that of the above four MLX preparations, MLX/HP-β-CD-ISG had the best-sustained release effect, and MLX-ISG and MLX/HP-β-CD had a more obvious sustained release effect than the MLX solution. The mathematical model fitting results of MLX release kinetics of different dosage forms were in the supplementary materials (Table S5, Supplementary Materials). The cumulative release rate data of MLX/HP-β-CD-ISG conformed to the Higuchi model, and in vitro dissolution linear correlation coefficient R^2^ was 0.9767, indicating that the Fickian diffusion fits the in vitro drug release of MLX/HP-β-CD-ISG better and the fitting result was good. The drug was released from the gel by diffusion, a mechanism that was consistent with the observed differences in the release rate due to the presence of HP-β-CD. The in situ gels incorporating HP-β-CD showed higher release rates, which remained stable [30]. These results agreed with those published by other authors [31,32], which further demonstrates that the incorporation of cyclodextrins in gel formulations can delay the release of a drug over time.

### 2.7. Pharmacokinetic Studies

**Figure 9:**
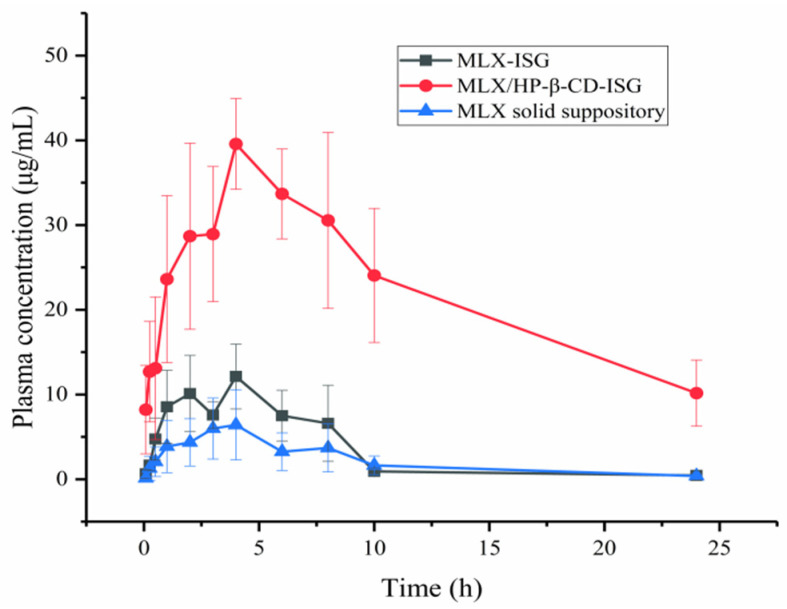
Plasma concentration-time profile of MLX concentration after the rectal administration of the MLX formulation. The results are expressed as X *±* SD (*n =* 6).

The rectal absorption of MLX was evaluated when MLX/HP-β-CD-ISG was administered via the rectum. The mean plasma concentration versus the time curves of MLX after the rectal administration is shown in Figure 9. The pharmacokinetic parameters are shown in Table 1. The plasma concentration of MLX in MLX/HP-β-CD-ISG was significantly higher than that of the MLX-ISG and MLX solid suppository at each observing time. According to the C_max_ and AUC values, MLX/HP-β-CD-ISG could achieve faster absorption and higher serum levels. The pharmacokinetic studies showed that, compared with the MLX solid suppositories, MLX/HP-β-CD-ISG successfully increased the bioavailability of MLX by about 5.28 times and t_1/2_ to about 5.98 h. Compared with MLX-ISG, after the rectal administration of MLX/HP-β-CD-ISG, the t_1/2_ and MRT of MLX were prolonged, and the AUC_(0–∞)_ was significantly increased (*p* < 0.01), indicating that MLX/HP-β-CD-ISG could improve the drug delivery at the administration site. The reason for this difference may be that the mucoadhesive gel matrices of P407 and P188 were formed at body temperature. The improved bioavailability of MLX could be attributed mainly to the avoidance of the hepatic first-pass effect, which was a consequence of drug retention in the lower rectum, with the aid of the polymer. The results suggest that MLX/HP-β-CD-ISG can be useful for MLX delivery, allowing easy self-administration by patients and avoiding the first-pass effects.

### 2.8. Rectal Retention Test

After the rectal administration of MLX/HP-β-CD-ISG containing methylene blue dye, its retention in the rectum was observed. After 0.5 h of administration, it becomes a dark blue gel. After 6 h, the blue gradually faded. Adhesion to the upper rectum in the form of a light blue gel occurred 12 h after administration. There was no obvious leakage in each period after administration, and they were distributed in the intestinal segment about 2~13 cm above the anus, with a wide distribution area. This means that the in situ gel is retained in the rectum for at least 12 h to ensure its release (Figure S1, Supplementary Materials).

### 2.9. Rat rectal Mucosal Irritation

**Figure 10:**
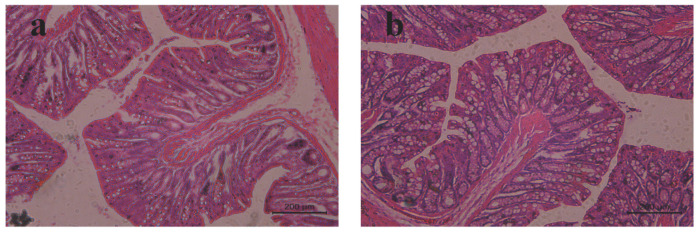
The pathological changes in the rectal tissue of rats in each group after rectal administration (HE × 200): (**a**) blank control group and (**b**) MLX/HP-β-CD-ISG rectal administration for 24 h.

The safety test was performed to observe any irritation or damage to rectal tissues in rats after the rectal administration of MLX/HP-β-CD-ISG. There was no abnormality in the rectal tissue observed by the naked eye. After histopathological sections were stained, the local mucosal epithelium of the rectum in the blank control group and the MLX/HP-β-CD-ISG group was intact, and there was no obvious inflammatory cell infiltration, edema or ulcer (Figure 10). This showed that MLX/HP-β-CD-ISG had no irritation or damage to the rectum.

## 3. Materials and Methods

### 3.1. Materials

MLX (content 99.6%) and piroxicam were purchased from Jinan Hongfangde Co., Ltd. (Shandong, China). Poloxamer 407 (P407) and Poloxamer 188 (P188) were purchased from BASF (Ludwigshafen, Germany). HP-β-CD was provided by Xi’an Deli Biochemical Co., Ltd. (Shanxi, China). Ammonium acetate, ammonia water, potassium dihydrogen phosphate and sodium hydroxide were obtained from Xilong Science Co., Ltd. (Shenzhen, China). Methylene blue (MB) was purchased from Shanghai Qingxi Chemical Technology Co., Ltd. (Shanghai, China). Purified water was used after deionization and filtration in a Millipore VR system. Dialysis membrane (MWCO 3.5 kDa) was purchased from Nanjing Senbega Biotechnology Co., Ltd. (Nanjing, China). Paraformaldehyde solution (4% PFA) was purchased from Shanghai Titan Scientific Co., Ltd. (Shanghai, China). Rhamsan gum was provided by Shanghai Yiyang Instrument Co., Ltd. (Shanghai, China). Hematoxylin and Eosin stain were purchased from Phygene Biotechnology Co., Ltd. (Fuzhou, China). Sliced paraffin (58~60 ℃) was purchased from Sinopharm Chemical Reagent Co., Ltd. (Shanghai, China). Acetonitrile and methanol of HPLC grade were purchased from Fisher Scientific (Waltham, MA, USA). All other chemical reagents and solvents used were of analytical grade.

### 3.2. Animals

In vivo studies were conducted using male Sprague-Dawley rats weighing 200~230 g. The experimental animals purchased from Silaikejingda Laboratory animals Co., Ltd., Changsha, China. The animal quality license number was SCXK 2019-0004. All experimental procedures in this study were conducted in accordance with the ethical principles of the use of experimental animals mandated by the State Key Laboratory (Reference number: BCTG-2016-18).

### 3.3. Screening of the Preparation Methods of Inclusion Compounds

MLX/HP-β-CD inclusion complexes were prepared by two methods with the MLX inclusion efficiency as the evaluation index. The specific operations were as follows: (1) Grinding method: An appropriate amount of HP-β-CD was placed in a mortar and dissolved in a wetting agent (1:1 water:methanol, *v*/*v*) to form a paste. In addition, an appropriate amount of MLX was added to the above paste, fully ground for 1 h, and then evaporated under negative pressure to remove moisture, then, the filter cake was dried to a constant weight in an oven at 60 °C. (2) Saturated solution method: HP-β-CD and MLX were completely dissolved in anhydrous ethanol and 25% ammonia under magnetic stirring, respectively. Then, the two solutions were mixed and stirred for 1 h and then evaporated at 75 °C until dry.

Three portions of MLX and HP-β-CD were accurately weighed, and the ratio of the two was 1:8. The appropriate amount of MLX/HP-β-CD prepared by the grinding method and saturated solution method was placed in a 10mL volumetric flask. After the ultrasonication of methanol for 20 min, it was diluted to the scale and shaken well. The sample was injected and determined under chromatographic conditions using the HPLC method [33]. The content of MLX was calculated by the external standard method, and the inclusion rate was calculated. The inclusion rate equation for MLX was as follows:

(2) $$\mathrm{Inclusion}\;\mathrm{Efficiency}\;(\%)=\frac{A}{B}\;\times \;100\%$$

where A is the MLX content in the inclusion complex and B is the MLX addition amount.

### 3.4. Optimization of the Preparation Method of the MLX/HP-β-CD Complexes

An MLX/HP-β-CD inclusion complex was prepared by the saturated solution method. According to the literature and preliminary experiments, it was found that the adding quantity of ammonia water (A), the ratio of MLX to HP-β-CD (B), inclusion time (C) and inclusion temperature (D) had a great influence on the inclusion interaction. Therefore, with the inclusion efficiency as the evaluation index, and the ratio of MLX to HP-β-CD (A), added quantity of ammonia water (B), inclusion time (C) and inclusion temperature (D) as the factors, three levels of each factor were optimized by the orthogonal test.

### 3.5. Characterization of the MLX/HP-β-CD Complexes

#### 3.5.1. Solubility

The solution experiment is the experimental method design of reference [34]. An excess of MLX (200 mg) and MLX/HP-β-CD inclusion complex (equivalent to 200 mg MLX) were placed in a 100 mL weighing bottle; then, 40 mL distilled water was added and stirred at 25 °C for 24 h to reach equilibrium (Figure S2, Supplementary Materials). After reaching equilibrium, the suspension was centrifuged at 13,000 r/min for 10 min, and the supernatant was filtered through a 0.45 μm filter membrane. The content of MLX in the filtrate was determined by HPLC, and the solubilities of the MLX and MLX/HP-β-CD inclusion complex were calculated.

#### 3.5.2. Phase Solubility

The study of phase solubility was a commonly used technique to analyze the inclusion complex stoichiometry [35]. According to the reported method, the phase solubility of MLX in the HP-β-CD aqueous solution was studied. In short, the gradient concentrations of HP-β-CD in distilled water (0, 0.02, 0.04, 0.06, 0.08 and 0.10 mmol/L) were first prepared. An excess of MLX was added to each HP-β-CD solution, and the sample was oscillated on a vortex for 1 min and then placed in a 37 °C thermostatic shaker at 200 r/min for 48 h. Equal samples were taken out and filtered with a 0.45 μm filter membrane. The concentration of MLX in the solution was determined by HPLC, and the results were drawn into a phase solubility diagram.

#### 3.5.3. Differential Scanning Calorimetry (DSC)

Differential scanning calorimetry (DSC) was performed on a Perkin-Elmer DSC 4000 to scan the powder of MLX, HP-β-CD, MLX/HP-β-CD inclusion complex and their physical mixture to analyze the inclusion of host and guest molecules. The specific conditions were as follows: the temperature range was 30~400 °C, the heating rate was 20 °C/min, and the nitrogen flow rate was 50 mL/min.

#### 3.5.4. Scanning Electron Microscope (SEM)

The surface morphology of MLX, HP-β-CD, physical mixtures and MLX/HP-β-CD complexes were examined by Jeol scanning JCM-7000 NeoScope (Tokyo, Japan). The surface of the samples for SEM was previously made electrically conductive in a sputtering apparatus (Fine coat ion sputter JFC-1100) by the evaporation of gold. The pictures were then taken at an excitation voltage of 20 kV.

#### 3.5.5. Powder X-ray Diffraction (PXRD)

XRPD spectra were obtained using a D8 ADVANCE diffractometer (Brukey, Germany). Powders of MLX, HP-β-CD, MLX/HP-β-CD inclusion complexes and their physical mixture were analyzed by X-ray diffraction. Test conditions were as follows: Cu as the target, graphite as the monochromator, a voltage of 44 kV, current of 30 mA, scanning rate of 4°/min, scanning range (2θ) of 10~80°.

#### 3.5.6. Fourier Transform Infrared Spectroscopy (FTIR)

FTIR spectrophotometry was carried out using a Thermo Nicolet iS5 instrument (Thermo, Waltham, MA, USA). Fourier transform infrared spectroscopy was used to scan the spectra of MLX, HP-β-CD, MLX/HP-β-CD inclusion complex and their physical mixture powder, with a wave number range of 400–4000 cm^−1^ and resolution of 2 cm^−1^.

### 3.6. Preparation of the Rectal In Situ Gel of MLX/HP-β-CD Inclusion Complex

#### 3.6.1. Preparation of the In Situ Gel and Determination of the Gelation Temperature

The weighed, prescribed amounts of P407 and P188 were placed in a beaker, and a certain amount of purified water was added to disperse them evenly. The clear and transparent, blank in situ gel was prepared by sealing and storing it in a refrigerator at 4 °C for more than 24 h. Then, the MLX/HP-β-CD inclusion complex was added under continuous stirring, and fully stirred evenly.

The commonly used temperature measurement methods include the magnetic stirring method and the tilt method [36]. The gelation temperature was measured by the tilt method. The thermometer was placed in a test tube containing 3 mL gel in a thermostatic water bath. The temperature was slowly increased, and the tube was tilted every 30 s to observe whether the gelation flowed. When the gel solution did not flow, the temperature in this state was the gelation temperature. Each sample was measured three times in parallel.

#### 3.6.2. Investigation of the Ratio of P407 and P188

P407 and P188 were used as temperature-sensitive materials for single-factor investigation. The thermosensitive gels with P407 mass concentrations of 18%, 19%, 20%, 21%, 23%, 24% and 25% were prepared and the gelation temperature was measured. Because P188 cannot complete the phase transition at a low concentration, and even if it completes the phase transition at a certain concentration, the required temperature still needs to be about 50 °C, so, in this experiment, P188 was only used to regulate the gelation temperature, and P407 was used as the matrix of the temperature-sensitive gel. Then, 2%, 4%, 6% and 8% P188 were added to the 18% P407 solution to prepare different gel solutions, and the gelation temperature was measured.

#### 3.6.3. Measurement of the Gelation Temperature and Gelation Time

The gelation temperature of the gel was measured by the above method. Another gel was placed in a 5 mL penicillin bottle and then placed in a water bath with 37 °C constant temperature. The change in the sample state was observed, and the time required for the sample to reach the gelation temperature was recorded.

#### 3.6.4. Measurement of Gel Strength

The gel strength was determined by Yong et al. [37]. First, 50 g gel was weighed and placed in a 100 mL graduated cylinder, which was placed in a 37 °C water bath. The gel strength tester with a weight of 35 g was placed in the gel solution. The gel strength was determined by the time (s) the apparatus took to sink 5 cm down through the gel. In cases where it took more than 300 s to drop the apparatus into the gel, various weights were placed on the top of the apparatus, and the gel strength was described by the minimal weights that pushed the apparatus 5 cm down through the gel.

#### 3.6.5. Measurement of the Viscosity

The viscosity of the gel samples was determined by NDJ-9S digital viscometer. An appropriate amount of gel was placed in a 50 mL beaker. According to the preliminary experimental results, the No. 3 rotor and rotation speed of 12 r/min were selected to determine the viscosity of the sample at room temperature and gelation temperature. Each sample experiment was repeated three times.

#### 3.6.6. Measurement of pH

Because the rectal administration preparation directly acts on the surface of the rectal mucosa, the pH value and mucosal irritation must be considered. In order to avoid irritation of the rectal mucosa, MLX/HP-β-CD-ISG at 34 °C was taken in a beaker and measured with a laboratory pH meter (Mettler Toledo, Budapest, Hungary).

#### 3.6.7. Measurement of the Rheological Properties

An Anton Paar Physica MCR101 rheometer (Anton Paar, Graz, Austria) was used, and a PP50 stainless steel parallel plate with a diameter of 50 mm and a gap of 1 mm was used [38]. Except for the determination of the phase transition temperature, other experiments were carried out at 35 ± 0.5 °C. Firstly, the linear viscoelastic region of the sample was measured. The maximum deformation that the three-dimensional network structure inside the gel can withstand was obtained by the test results. The linear viscoelastic region of the sample was measured by amplitude scanning (strain was 0.01~100% and frequency was 10 rad/s). Then, the gel was frequency scanned in the oscillation mode (strain was 1%, frequency scanning range was 0.1~100 Hz). Finally, the phase transition temperature of gel was measured in the oscillating mode. The frequency was fixed at 1 Hz and 1% strain was performed. The sample was heated at a rate of 2 °C/min and the temperature range was 4~50 °C.

#### 3.6.8. Measurement of Stability

MLX/HP-β-CD-ISG was placed in different environments [39,40], including low temperature (4 °C), high temperature (40 °C), high humidity (25 °C, relative humidity 90% ± 5%) and high-intensity light 4500 lx ± 500 lx. The changes of MLX content, pH value and gel temperature in MLX/HP-β-CD-ISG at 0, 5 and 10 d were investigated to determine the optimal storage conditions.

### 3.7. In Vitro Drug Release Study

In vitro release studies were carried out by using the dialysis method [41]. In 500 mL phosphate buffer solution (pH 7.4 PBS), at 50 rpm and 37 ± 0.5 °C, the preparation was placed into a dialysis bag (MWCO = 8~14 kD) boiled in boiling water, soaked for 24 h and stirred in a dissolution tester (Vision Classic 6, Hanson, KY, USA). At a predetermined time interval, an aliquot sample (1 mL) was taken from the released dissolution medium and replaced with freshly prepared phosphate buffer solution. The content of MLX was determined by HPLC. All experiments were conducted in quadruplicate, and the drug release mechanism of the preparation was discussed by using three commonly used mathematical models: zero-order, first-order kinetics and the Higuchi model.

### 3.8. Pharmacokinetic Study

#### 3.8.1. HPLC Method

The quantification of MLX in formulation samples was determined by a validated reversed-phase HPLC-UV method (Shimdzu, Japan). The HPLC system consisted of an LC-20AT pump, an SPD-M20A PAD-visible detector, a SIL-20A injector with a 20 μL loop and an LC solution workstation.

The separation was performed using an Elite Hypersil ODS2 column (4.6 mm × 250 mm, 5 μm). The mobile phase was methanol: 0.2% ammonium acetate aqueous solution (50:50, *v*/*v*); flow rate: 0.8 mL/min; detection wavelength: 360 nm; injection volume: 10 μL, and the column temperature was 37 °C.

Following HPLC separation, the content of MLX was measured by the peak-area ratio of MLX and the piroxicam method.

To determine the standard calibration curve, serial dilutions of MLX in plasma were prepared (50, 40, 30, 20, 10, 4 and 1 μg/mL). The peak areas of MLX and piroxicam were recorded for the different concentrations of MLX, and linear regression was performed on the concentration (C) by the ratio of the two to obtain the standard curve. A linear regression equation was determined (R^2^ = 0.9918) and used to calculate the MLX amount in subsequent experiments (Figure S3, Supplementary Materials). The calibration curves for MLX in rat plasma were linear in the concentration range of 1~50 μg/mL with correlation coefficients (R^2^) > 0.9918. The lower limit of quantification (LLOQ) was defined as the lowest standard concentration on the calibration curve with an accuracy of 90–115 % and a precision less than 15 %. The LLOQ of MLX was 1 μg/mL, and the limit of detection (LOD) was 1 ng/mL, which provided sufficient sensitivity to characterize pharmacokinetics.

#### 3.8.2. Grouping and Dosing

A total of 18 SD rats weighing 180~220 g were randomly divided into 3 groups (MLX-ISG group, MLX solid suppository group and MLX/HP-β-CD-ISG group) for rectal administration, with 6 rats in each group. Each group was administered a dose of 15 mg/kg and 1 mL of the solutions.

#### 3.8.3. Collection and Treatment of the Plasma Samples

0.5 mL blood samples were collected at 0.083, 0.25, 0.5, 1, 2, 3, 4, 6, 8, 10 and 24 h after administration. The collected blood samples were placed in a pre-heparinized 1.5 mL anticoagulant centrifuge tube, centrifuged at 4 °C, 8000 rpm/min for 10 min, and the supernatant was drawn into another centrifuge tube. Cryopreservation was conducted at −80 °C. After the plasma sample was thawed at room temperature, 100 μL of plasma, 200 μL of acetonitrile and 100 μL of internal standard solution (2.5 μg/mL piroxicam) were accurately weighed in a centrifuge tube and vortexed for 3 min to precipitate the proteins. After centrifugation at 13,000 rpm for 10 min at 4 °C, the supernatant was filtered through a 0.22 μm membrane, and the plasma drug content was determined according to the above HPLC method. The obtained MLX plasma concentration results were processed according to the DAS 3.0 program non-compartmental model. (Beijing JiDaoChengran Technology Co., Ltd., Beijing, China).

### 3.9. Rectal Retention Test

Three male SD rats were taken. According to the reference [42], 0.1% methylene blue dye was added to the prescription, and 1 mL gel was passed through the anus through a hose to the rectum 2 cm above the anus. The hair color around the anus was observed at 0.5, 3, 6 and 12 h after administration to determine the leakage. Then, three rats were sacrificed, and the distribution and adhesion of the gel in the rectum were observed.

### 3.10. Rat Rectal Mucosal Irritation

Twelve SD rats, weighing 200~220 g, were randomly divided into two groups: the MLX/HP-β-CD inclusion complex in situ gel group and a blank control group. Before the experiment, the rectal health of the rats was observed, and no congestion, swelling and ulcer were observed. The animals were fasted for 12 h before drug administration, with free water drinking being allowed. During this period, 2 mL warm boiled water was drawn and injected into the anus of rats, about 4 cm away, to promote the emptying of their feces (twice). The blank group did not have any treatments. The MLX/HP-β-CD inclusion complex in situ gel group was administered 0.5 mL of the gel. After administration, it was inverted to prevent its outflow and maintained for 2 min, so that the drug was in full contact with the intestinal mucosa. After 24 h, the anus was observed for edema, congestion or discharge of secretions. After the intraperitoneal injection of urethane, the rectum was taken and fixed in 4% paraformaldehyde for over 24 h. It was dehydrated, paraffin-embedded, sliced, stained and observed under a microscope (Leica DM2500, Wetzlar, Germany).

### 3.11. Statistical Analysis

All data were expressed in X ± SD. SPSS 26.0 software (International Business Machines Corporation, New York, NY, USA) was used for one-way ANOVA and pairwise comparisons with Tukey’s test or LSD test.

## 4. Conclusions

In this experiment, MLX was prepared as a rectal sustained-release preparation. Firstly, HP-β-CD was used as the material to encapsulate the drug MLX using the saturated solution method. The preparation conditions of the inclusion complex were optimized via the orthogonal experiment. The results showed that the encapsulation efficiency of the inclusion complex was the best when the added quantity of ammonia water was 15 mL, the ratio of MLX to HP-β-CD was 1:8, the inclusion time was 60 min and the inclusion temperature was 40 °C. The inclusion complex was identified by DSC, SEM, PXRD and FTIR. A new inclusion complex with good stability was formed by MLX and HP-β-CD. The preparation method was simple, feasible and reproducible. Then, the inclusion complex was further formulated into a rectal in situ gel. The optimized content of poloxamer 407 and poloxamer 188 in the final formulation was 18% and 4%, respectively. The results show that the in situ gel prepared using the optimal composition changed from the solution state to the semi-solid state at 33.40 ± 0.17 °C. From the in vitro release curve, it can be seen that the prepared gel released about 70% of the drug amount in about 10 h. The release process conformed to the Higuchi model and had a certain degree of sustained release effect. More importantly, MLX/HP-β-CD-ISG absorbed MLX better, improving its bioavailability in rats by increasing its retention time in the rectum. In addition, MLX/HP-β-CD-ISG produced no irritation of rectal tissue. These results suggested that MLX/HP-β-CD-ISG has potential application prospects as a rectal administration preparation.

The preparation method was simple, the stability was good, the sustained release effect was good, the rectum was not irritated, and the number of administrations could be reduced. It had a better solubility than MLX, increased the rectal retention time and improved its bioavailability, and is expected to become a more convenient and effective non-steroidal anti-inflammatory rectal administration.

## Figures and Tables

**Table 1:** Pharmacokinetic parameters of MLX after rectal administration (X ± SD, *n* = 6).

Parameter	MLX-ISG	MLX/HP-β-CD-ISG	MLX Solid Suppository
t_½_ (h)	4.12 ± 3.02	10.62 ± 4.76	4.64 ± 1.44
T_max_ (h)	2.83 ± 1.33	4.50 ± 2.17	3.33 ± 0.52
C_max_ (µg/mL)	13.57 ± 3.68 **	40.68 ± 4.89 **##	7.70 ± 4.030 ##
AUC_(0→t)_ (µg·h/mL)	77.08 ± 19.04 **	491.44 ± 127.50 **##	47.38 ± 23.48 ##
AUC_(0→∞)_ (µg·h/mL)	81.98 ± 15.90 **	387.77 ± 134.21 **##	51.52 ± 21.97 ##
MRT_(0→∞)_ (h)	7.04 ± 4.62	16.79 ± 7.29	12.130 ± 12.02

Notes: ** *p <* 0.01 vs. MLX-ISG; ## *p <* 0.01 vs. MLX solid suppository; t_½_, elimination half-life; T_max_, time to reach peak plasma concentration; C_max_, peak plasma concentration; AUC_(0→∞)_, area under the plasma concentration-time curve calculated by the trapezoidal rule from time 0 to infinity; SD, standard deviation.

## Data Availability

Data are contained within the article and Supplementary Materials.

## Supplementary Materials

The following supporting information can be downloaded at: https://www.mdpi.com/article/10.3390/molecules28104099/s1, Figure S1: In vivo localization of MLX/HP-β-CD-ISG in the rectum at (a) 30min; (b) 3h; (c) 6h; and (d) 12h after rectal administration. Figure S2: Standard curve and linear range. Figure S3: Standard curve and linear range. Table S1: Factors and levels. Table S2: Orthogonal design results. Table S3: Analysis of variance. Table S4: Stability investigation of MLX/HP-β-CD-ISG. Table S5: The mathematical model fitting of release kinetics of MLX from different formulations.

## Sample Availability

Samples of the compounds are not available from the authors.
