# Supplementary material for: Preparation and In Vitro and In Vivo Evaluation of Rectal In Situ Gel of Meloxicam Hydroxypropyl-β-cyclodextrin Inclusion Complex
Source: Molecules. 2023 May 15;28(10):4099. doi: 10.3390/molecules28104099 (PMC10223448; doi:10.3390/molecules28104099)
Supplement: Supplementary file 1 [file molecules-28-04099-s001.zip › molecules-2268437-supplementary.pdf]

Supplementary materials

Tables

Table S1 Factors and levels.

Level	Factor			
	adding quantity of ammonia water (A)	the ratio of MLX to HP-β-CD (B)	inclusion time (C)	inclusion temperature (D)
1	5	1:4	30	20
2	10	1:6	60	30
3	15	1:8	90	40

Table S2 Orthogonal design results.

Number	A	B	C	D	Inclusion Rate
1	1	1	1	1	33.59±0.93
2	1	2	2	2	71.63±2.58
3	1	3	3	3	86.24±3.32
4	2	1	3	2	56.39±2.95
5	2	2	1	3	71.34±0.97
6	2	3	2	1	79.86±1.31
7	3	1	2	3	57.31±2.49
8	3	2	3	1	88.85±1.36
9	3	3	1	2	78.61±0.53
K1	63.82	47.00	61.18	67.43	
K2	68.30	77.27	68.40	67.98	
K3	73.72	81.57	66.26	70.43	
R	9.90	34.57	15.08	3.00	

Table S3 Analysis of variance.

Error source	SS	f	S	F	P
A	147.57	2	73.78	9.65	>0.05
B	2130.45	2	1065.23	139.30	<0.05
C	15.29	2	7.65		>0.05
D	341.31	2	170.66	22.32	>0.05

Table S4 Stability investigation of MLX/HP-β-CD-ISG.

		0d	5d	10d
4°C	MLX content	100%	97.32%	95.49%
	pH	7.12±0.05	6.95±0.01	6.66±0.02
	Gelation Temperature	(33.40±0.17)°C	(33.10±0.1)°C	(33.80±0.2)°C
high light	MLX content	100%	95.01%	83.97%
	pH	7.12±0.05	7.10±0.02	7.07±0.03
	Gelation Temperature	(33.40±0.17)°C	(33.50±0.2)°C	(33.20±0.3)°C

high humidity	MLX content	100%	87.36%	74.53%
	pH	7.12±0.05	6.67±0.03	6.53±0.05
	Gelation Temperature	(33.40±0.17)°C	(34.20±0.3)°C	(34.10±0.3)°C
high temperature	MLX content	100%	83.91%	70.82%
	pH	7.12±0.05	6.50±0.03	6.32±0.03
	Gelation Temperature	(33.40±0.17)°C	(33.10±0.3)°C	(34.50±0.2)°C

Table S5 The mathematical model fitting of release kinetics of MLX from different formulations.

**Model**	**MLX solution**	**MLX/HP-β-CD**	**MLX-ISG**	**MLX/HP-β-CD-ISG**
Zero-order kinetic	$M_t=2.68t+52.97$ $R^2=0.6179$	$M_t=3.28t+27.42$ $R^2=0.8040$	$M_t=3.26t+22.93$ $R^2=0.8426$	$M_t=3.06t+19.06$ $R^2=0.8588$
First-order kinetic	$\ln M_t=0.76t+92.57$ $R^2=0.8298$	$\ln M_t=0.24t+84.51$ $R^2=0.9496$	$\ln M_t=0.20t+82.16$ $R^2=0.9645$	$\ln M_t=0.18t+76.17$ $R^2=0.9753$
Higuchi kinetic	$M_t=16.10t^{1/2}+35.96$ $R^2=0.8333$	$M_t=18.28t^{1/2}+9.71$ $R^2=0.9628$	$M_t=18.08t^{1/2}+5.58$ $R^2=0.9709$	$M_t=16.87t^{1/2}+3.02$ $R^2=0.9767$

Figures

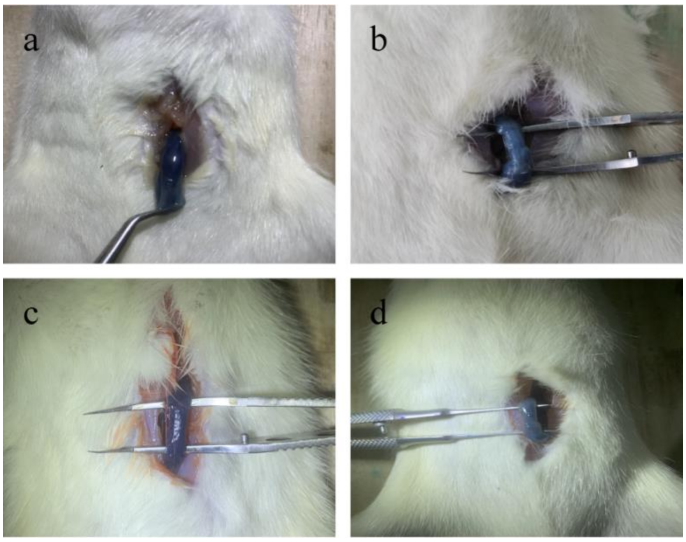

Figure S1 In vivo localization of MLX/HP-β-CD-ISG in the rectum at (a) 30min; (b) 3h; (c) 6h; and (d) 12h after rectal administration.

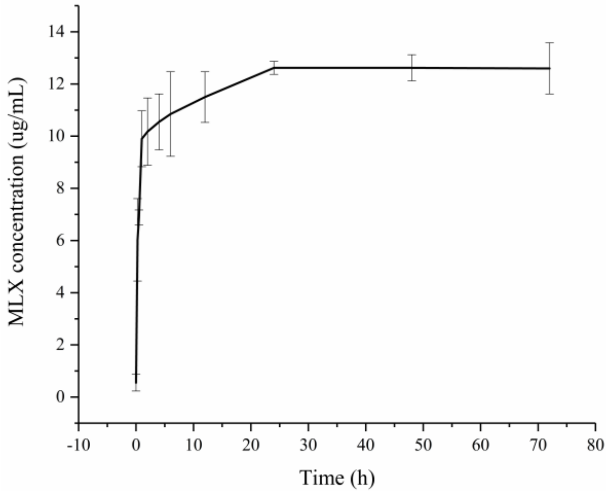

Figure S2 The equilibration time of MLX Solubility.

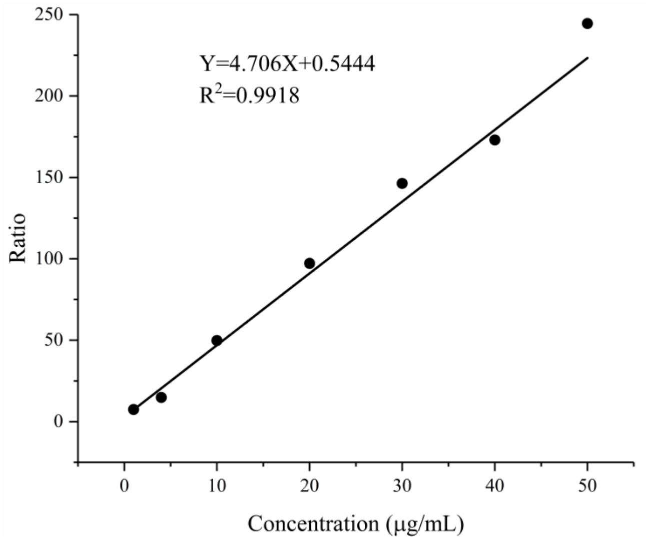

Figure S3 Standard curve and linear range.
